# The Clinical Application of Established and Emerging Biomarkers for Chronic Respiratory Diseases

**DOI:** 10.3390/jcm12196125

**Published:** 2023-09-22

**Authors:** Pasquale Ambrosino, Giuseppina Marcuccio, Fabio Manzo, Costantino Mancusi, Claudia Merola, Mauro Maniscalco

**Affiliations:** 1 Istituti Clinici Scientifici Maugeri IRCCS, Directorate of Telese Terme Institute, 82037 Telese Terme, Italy; 2 Istituti Clinici Scientifici Maugeri IRCCS, Pulmonary Rehabilitation Unit of Telese Terme Institute, 82037 Telese Terme, Italy; giuseppina.marcuccio@icsmaugeri.it (G.M.); claudia.merola@icsmaugeri.it (C.M.); 3 Fleming Clinical Laboratory, 81020 Casapulla, Italy; fabioce26@gmail.com; 4 Department of Advanced Biomedical Sciences, Federico II University, 80131 Naples, Italy; costantino.mancusi@unina.it; 5 Department of Clinical Medicine and Surgery, Federico II University, 80131 Naples, Italy

Biomarkers are indicators of a pathological or physiological state, and they are essential for facilitating the diagnosis of a subclinical condition, understanding the origin or progression of a disease, stratifying the risk, and assessing the response to a specific therapeutic approach [1]. Although the term is often overused, it can be argued that any molecular, histological, physiological, or radiological characteristic that can be objectively quantified can be considered a biomarker [2]. Biomarkers became an active part of medical research in the last decades of the 1900s and in recent years, there has been a rapid evolution of their application in medicine and biology [3]. Thus, as omics sciences continue to advance and as new and intricate molecular biology techniques are perfected, biomarkers have progressively emerged as stable milestones in the management of disease [4]. Some of them are consolidated in clinical practice, while others are to be counted among the emerging ones. To date, a growing number of novel biomarkers are under study and their use has been proposed in medicine and biology, in view of the high number of potential future applications in diagnosis, prognosis, risk stratification, and drug response monitoring [3].

In this context, there is increased attention being paid to in-depth research on biological and clinical markers for their potential in the management of chronic respiratory diseases (CRDs) [2,5], as they are experiencing worldwide increases in both their occurrence and associated mortality rates with enormous burden in terms of disability, social costs, and rehabilitation needs [6,7]. Considering the diverse nature of CRDs, prioritizing the identification of distinct biomarkers associated with the varied etiologies of individual conditions is imperative [3,5]. This approach aims to facilitate precise and personalized management by enabling early diagnosis and the implementation of suitable pharmacological and rehabilitation treatments to control the progression of each pathology [4].

A number of clinical and functional measures have been identified and are currently used in clinical practice as biomarkers of CRDs, along with various laboratory determinations conducted on serum, sputum, and bronchoalveolar lavage fluid [5]. The list of biomarkers in CRDs is extensive and continually evolving. However, it is important to emphasize that, while the tools available for rare pulmonary diseases (e.g., interstitial lung diseases, hypersensitivity pneumonia, cystic fibrosis) is still restricted and often limited to radiological imaging and invasive procedures [8], the clinician’s arsenal in more common clinical conditions is large, as it includes a wealth of conventional biomarkers. Although forced expiratory volume in 1 s (FEV_1_) is the most frequently utilized biomarker in chronic obstructive pulmonary disease (COPD), FEV_1_ decline is highly variable among COPD patients and airway limitation demonstrates only a partial correlation with symptoms and disease progression [9]. In this regard, the assessment of diffusing lung capacity for carbon monoxide (DLCO) has provided additional insights into the wide clinical spectrum of COPD, identifying emphysematous changes even before significant airflow limitation is evident [2]. Considering that COPD is an inflammatory disease, fibrinogen and C-reactive protein (CRP) often show elevation in patients experiencing frequent exacerbations, with them also being associated with all-cause mortality. However, their ability to predict future events and disease progression remains unclear [10]. With respect to this, the soluble receptor for advanced glycation end products (sRAGE) has been widely accepted as an additional marker of inflammation in COPD, having the potential to predict the progression of emphysema [11]. In fact, due to its ability to intercept and neutralize pro-inflammatory molecules in the lungs, its blood levels have shown an inverse correlation with emphysema and airflow limitation [11]. Similar to sRAGE, other proteins originating from lung parenchymal cells, namely surfactant protein D (SP-D) and club cell protein 16 (CC16), have also been associated with decline in FEV_1_ [12,13]. Accordingly, the combination of CC16, fibrinogen, and sRAGE best predicted decline in lung function in large cohort studies [14].

Many other molecules in different biological fluids have demonstrated the characteristics of an ideal biomarker in CRDs, with the potential to predict disease progression and treatment response [5]. Although the concept of a clinical marker is closely related but not exactly overlapping with that of a biomarker [2], there is also a wide range of clinical measures in CRDs that are capable of predicting relevant clinical outcomes. Among these, the 6-min walking distance (6MWD) has been found to be a valuable tool for evaluating the functional status and mortality risk of CRD patients due to its ease of use, cost-effectiveness, and high potential for standardization [6]. Similarly, the modified Medical Research Council (mMRC) questionnaire and the COPD assessment test (CAT) have been effectively used in clinical guidelines and, consequently, in clinical practice for staging disease severity and predicting health status deterioration, exacerbations, and mortality, not only in COPD but also in other clinical settings [15]. Combining many of the aforementioned clinical and functional measures, the BODE index has progressively emerged as a composite clinical marker, with high prognostic value compared to other standard geriatric tools [16]. The BODE index effectively integrates four independent predictors, namely body mass index, FEV_1_, mMRC score, and 6MWD, thus providing a comprehensive and multidimensional assessment which is strongly related to mortality and even to the future utilization of health resources [2].

Alongside COPD, asthma stands as the most prevalent chronic condition, impacting approximately 14% of children worldwide, and its prevalence continues to increase [17]. Therefore, biomarkers may offer numerous applications in the investigation and treatment of asthma, with the potential to play a pivotal role in both identifying specific asthma endotypes and predicting therapeutic responses [18]. The total quantity of immunoglobulin E (IgE) and the presence of allergen-specific IgE antibodies in the blood are currently used as biomarkers for characterizing the phenotype of patients experiencing symptoms of allergic asthma [19]. Other biomarkers of T helper 2 (Th2) inflammation include blood and sputum eosinophiles, fractional exhaled nitric oxide (FeNO), and periostin, which are all predictive of airway eosinophilia, response to corticosteroids, and the risk of exacerbations [5]. Overall, these biomarkers of Th2 inflammation differentiate patients who exhibit distinct immune cell infiltration and histological alterations, thus defining a specific endotype of asthma which may respond to specific biological therapies. The fact that Th2 inflammation has emerged as a relevant pathogenic mechanism not only in asthma but also in up to 40% of COPD cases has marked a common immunological thread in these distinct respiratory conditions [20], providing further impetus for the exploration of novel biomarkers and new targeted therapies. In allergic asthma, allergen exposure activates Th2-mediated inflammation, which in turn coordinates an immune response characterized by the release of interleukin (IL)-4, IL-5, and IL-13. These cytokines are known to participate in airway eosinophilia, inflammation, and hyperresponsiveness, with mucus hypersecretion and parenchyma remodeling [21]. While allergen-specific Th2 cells drive the onset of asthma, non-specific innate lymphoid cells (ILC2s) appear to play a role in inducing eosinophilic inflammation in nonallergic asthma and COPD [22]. The relatively recent discovery of ILC2s holds the potential to establish a connection between innate and adaptive immune responses, particularly in CRDs characterized by type 2 inflammation [23]. A suggested hypothesis is that ILC2s in COPD patients could be activated by inflammatory mediators released by the pulmonary epithelium and other lung-specific cells. This cascade leads to the production of several cytokines, including IL-4, IL-5, and IL-13, which are recognized contributors to airway eosinophilia and parenchymal remodeling [24]. In the era of precision medicine, a better knowledge of these mechanisms has primarily led to the introduction of new biologic drugs capable of directly targeting IL-5 (mepolizumab and reslizumab), its receptor (benralizumab), or the alpha subunit of the IL-4 receptor (dupilumab), whose use has been considered not only in severe asthma but also eosinophilic COPD [25]. On the other hand, this has sparked growing interest in the development of non-invasive and rapid Th2 inflammatory markers. Although induced sputum has traditionally been regarded as the gold standard for assessing eosinophilic airway inflammation, there is substantial evidence of a strong correlation between FeNO levels, eosinophil counts in sputum, and eosinophil counts in blood [25]. Consequently, FeNO has gained widespread acceptance in routine clinical practice in recent years as a convenient surrogate marker for airway eosinophilia, primarily due to its non-invasive nature and ease of use, thus being routinely employed to support diagnosis and monitor response and adherence to steroids in asthma [5]. In contrast, a certain degree of FeNO variability has been recently documented in COPD, thus questioning its real usefulness as a biomarker of airway eosinophilia in this clinical setting [24].

A growing number of other specific, repeatable, and non-expensive biomarkers are on the horizon, making it realistic to imagine that this will change the management of all respiratory conditions in the future, especially rare pulmonary diseases for which the diagnostic and therapeutic options are still relatively limited [8]. In this regard, a striking example is represented by pulmonary arterial hypertension (PAH), whose management has significantly improved in recent years with the help of new clinical and biological markers. Hence, alongside some validated clinical scores such as REVEAL 2.0 [26], brain natriuretic peptide (BNP) and its N-terminal peptide (NT-proBNP) have seen growing utilization in risk assessment, treatment efficacy monitoring, and even PAH screening in individuals with systemic sclerosis [27]. Similarly, lung-specific proteins, such as SP-D and Krebs von den Lunghen-6 (KL6), are currently being explored as biomarkers of fibrosis and inflammation in nonspecific interstitial pneumonia (NSIP), idiopathic pulmonary fibrosis (IPF), hypersensitivity pneumonia, cryptogenic organizing pneumonia, and radiation pneumonitis [28,29]. A similar role as a nonspecific biomarker of extracellular matrix remodeling and fibroproliferation in IPF has been demonstrated for serum matrix metalloproteinase 7 (MMP7), which is currently being used in other clinical conditions characterized by progressive fibrosis, such as chronic liver diseases [5,29].

Recently, given the systemic nature of CRDs, growing interest has also been directed toward the use of biomarkers able to detect the early signs of comorbidities, particularly metabolic and cardiovascular comorbidities. While BNP and NT-proBNP are recognized indicators of ventricular stress [27], the study of endothelial function has emerged in recent times as a potential biomarker for arterial thrombotic risk in COPD and post-acute coronavirus disease 2019 (COVID-19) syndrome, thus allowing for an exploration of the concurrent impact of pharmacological and rehabilitation strategies on this risk [30,31]. Similarly, considering that systemic inflammation is a common pathogenic background in CRDs, the simultaneous elevation of CRP, fibrinogen, and leukocyte count has been regarded as a composite marker of the risk of myocardial infarction, diabetes, and heart failure in individuals with COPD [32].

Overall, it is currently difficult to provide a complete and exhaustive overview of all known and under-studied biomarkers in CRDs. In this context, it is important to remember that omics technologies, such as metabolomics, genomics, and proteomics, will probably revolutionize our approach to CRDs, with them holding promise for uncovering novel therapeutic targets and optimizing pharmacological and rehabilitation interventions. Concerning this matter, further preclinical, translational, and clinical research will provide insights into genetic predispositions, molecular pathways, and biomarkers that can improve diagnosis, personalized treatment, and disease management.
